# Accelerated Development of COVID-19 Vaccines: Technology Platforms, Benefits, and Associated Risks

**DOI:** 10.3390/vaccines9070747

**Published:** 2021-07-06

**Authors:** Ralf Wagner, Eberhard Hildt, Elena Grabski, Yuansheng Sun, Heidi Meyer, Annette Lommel, Brigitte Keller-Stanislawski, Jan Müller-Berghaus, Klaus Cichutek

**Affiliations:** Paul-Ehrlich-Institut, Federal Institute for Vaccines and Biomedicines, 63225 Langen, Germany; ralf.wagner@pei.de (R.W.); Eberhard.Hildt@pei.de (E.H.); elena.grabski@pei.de (E.G.); Yuansheng.sun@pei.de (Y.S.); Heidi.Meyer@pei.de (H.M.); Annette.Lommel@pei.de (A.L.); brigitte.keller-stanislawski@pei.de (B.K.-S.); Jan.Mueller-Berghaus@pei.de (J.M.-B.)

**Keywords:** SARS-CoV-2, vaccine development, clinical trials, marketing authorization

## Abstract

Multiple preventive COVID-19 vaccines have been developed during the ongoing SARS coronavirus (CoV) 2 pandemic, utilizing a variety of technology platforms, which have different properties, advantages, and disadvantages. The acceleration in vaccine development required to combat the current pandemic is not at the expense of the necessary regulatory requirements, including robust and comprehensive data collection along with clinical product safety and efficacy evaluation. Due to the previous development of vaccine candidates against the related highly pathogenic coronaviruses SARS-CoV and MERS-CoV, the antigen that elicits immune protection is known: the surface spike protein of SARS-CoV-2 or specific domains encoded in that protein, e.g., the receptor binding domain. From a scientific point of view and in accordance with legal frameworks and regulatory practices, for the approval of a clinic trial, the Paul-Ehrlich-Institut requires preclinical testing of vaccine candidates, including general pharmacology and toxicology as well as immunogenicity. For COVID-19 vaccine candidates, based on existing platform technologies with a sufficiently broad data base, pharmacological–toxicological testing in the case of repeated administration, quantifying systemic distribution, and proof of vaccination protection in animal models can be carried out in parallel to phase 1 or 1/2 clinical trials. To reduce the theoretical risk of an increased respiratory illness through infection-enhancing antibodies or as a result of Th2 polarization and altered cytokine profiles of the immune response following vaccination, which are of specific concern for COVID-19 vaccines, appropriate investigative testing is imperative. In general, phase 1 (vaccine safety) and 2 (dose finding, vaccination schedule) clinical trials can be combined, and combined phase 2/3 trials are recommended to determine safety and efficacy. By applying these fundamental requirements not only for the approval and analysis of clinical trials but also for the regulatory evaluation during the assessment of marketing authorization applications, several efficacious and safe COVID-19 vaccines have been licensed in the EU by unprecedentedly fast and flexible procedures. Procedural and regulatory–scientific aspects of the COVID-19 licensing processes are described in this review.

## 1. Introduction

SARS-CoV-2 has been described in late 2019 as the pathogen associated with a new syndrome in Wuhan, China, which is now termed SARS-CoV-2-related coronavirus disease (COVID-19). The virus has subsequently spread globally due to high transmissibility between humans, with far-reaching consequences. The pandemic has led to excessive overburdening of many healthcare systems globally, including high numbers of hospitalizations and deaths [1]. Worldwide, more ventilation beds than those available were often required for the intensive care treatment of patients severely ill with COVID-19. During the peak time of the pandemic, the number of COVID-19-related deaths continued to rapidly increase, and still today cases of infections, diseases, and deaths are perpetually detected. Public health countermeasures, including full ‘lockdowns’, aim to reduce infection rates; however, these measures are having huge negative impacts on social activities and the economies of the affected countries. Novel vaccines have evolved as valuable tools to protect against COVID-19.

Vaccines offer the most effective solution to resolve the current pandemic due to (i) the prevention of virus-induced COVID-19 (or at least of severe cases) and (ii) the reduction of human-to-human transmission rates. According to the WHO, over 270 vaccine projects have been started worldwide, with almost 100 clinical trials of specific COVID-19 vaccines initiated until the first quarter of 2021 [2]. This extremely high number of product developments reflects the urgent need for different COVID-19 vaccines, the high production capacity of doses necessary for global use, as well as the medical urgency and the need to return to a normal life without contact restrictions.

A multitude of established and experimental vaccine platforms have been developed for COVID-19 prevention, including platforms based on genetic information such as DNA, RNA, and vector vaccines, as well as subunit vaccines based on genetically engineered protein antigens, peptide vaccines, and even adjuvanted inactivated whole virus vaccines [3].

Due to the urgent need, vaccine development has been given the highest priority not only by political decision-makers but also by the academia, pharmaceutical industry, and medicines regulatory agencies. However, despite this urgent need, it is of crucial importance to ensure the necessary care and scrutiny in regulation and product evaluation. Worldwide, agreements have been made between the globally active medicines regulatory agencies on the criteria for the approval of clinical trials. Creating the necessary and appropriate balance between possible regulatory simplifications and essential requirements is a scientific task, the basics of which the Paul-Ehrlich-Institut, as the Federal Institute for Vaccines and Biomedical Medicines, has summarized in the following text.

A few basic conditions are outlined: (i) vaccine development is always the development of a specific vaccine product, (ii) vaccine products are subject to approval, in Germany only by the Paul-Ehrlich-Institut, in all European Member States by the European Commission, (iii) the approval is granted for a particular vaccine product as specified in the marketing authorization with its dedicated defined indication, e.g., preventive vaccination for persons of a certain age group or other characteristics, (iv) in Germany, the actual use of the vaccine in certain groups of persons in accordance with the approval is recommended by the Standing Vaccination Recommendation Committee at the Robert Koch-Institut, which can also be adopted as a vaccination recommendation by the state medical associations. This provides the foundation for a health economics evaluation by the Federal Joint Committee, including price setting and the reimbursement by health insurance companies.

As of 02 June 2021, four COVID-19 vaccines have been granted a conditional approval (see below) in the EU, as presented in Table 1 below [4].

## 2. Vaccine Quality-Related Regulatory Requirements

As for common vaccines, the establishment of a fully quality-assured manufacturing process is of fundamental importance for COVID-19 vaccine development. This requires detailed process-specific developments and specifications as well as the implementation of suitable control measures including in-process controls. The entire production of vaccines must meet the requirements of “Good Manufacturing Practice” (GMP) [5], which is certified by a manufacturing permit (usually issued after an official on-site inspection of the manufacturing facility). In Germany, the state authority in whose regional area the manufacturing facility is located grants this permit in consultation with the Paul-Ehrlich-Institut. For this purpose, the consistency of production is typically proven through three consecutive production processes for an identical product. A certain degree of flexibility has been applied for COVID-19 vaccines as regards the manufacturing range of these process performance qualification lots or by taking into account the respective data for similar products of the same platform technology. From a regulatory perspective, the quality module (which has to be submitted with the application for approval of a clinical trial as well as with the application for marketing authorization) forms the basis for ensuring the consistent and impeccable pharmaceutical quality of the vaccine produced. The quality documents contain detailed descriptions of the entire process steps with all relevant intermediate stages/products and complete information on the nature and origin of all raw materials. The quality requirements for vaccines are legally defined in Annex 1 to the European Directive 2001/83/EC [6].

Particular attention during production is given to materials with biological origin, such as cell substrates for virus cultivation or protein expression, seed viruses, and media components. This also includes all active enhancers (adjuvants) or special formulation components and stabilizers used. Furthermore, all implemented control processes including the specifications established for release testing, which were implemented to ensure the required and consistent vaccine quality, must be precisely described and justified. For licensure, the manufacturing process and the control methods used must be validated. The permissible target ranges and acceptance criteria for the control tests should be based on the experimental data generated in the course of development and during process validation. These tests also include controls and the minimization of any possible contamination. Essential components of the quality module relate to the description of the cultivation systems and the measures that have been taken to reliably exclude any microbial, in particular viral, contamination.

For the end product, it must be ensured that the active ingredient content is constant and that a homogeneous formulation of the end product is guaranteed. All other quality-determining parameters must also be checked and confirmed. This is ensured by intensive testing of the end product (prior to the official release) by the manufacturer in accordance with the test program established as part of the approval process. Approved vaccines are also subject to batch testing by the Paul-Ehrlich-Institut or another official control laboratory from the European OMCL network. This ensures that release tests of particular importance for quality assurance, such as potency, must undergo an additional experimental check by a qualified public test laboratory before the official batch release for the market. In line with this general requirement, all COVID-19 vaccine lots on the European market are experimentally tested and officially released by a European OMCL.

For the practical use of the vaccine after release, the shelf life under the predefined storage conditions is a decisive factor. Therefore, the results of the vaccine-specific stability and shelf-life studies carried out by the manufacturer are an important part of quality documentation, on the basis of which the shelf life is defined and approved at the respective development stage. For the very rapidly developed COVID-19 vaccines, only preliminary and short-termed stability results were available at the time of licensure, as the storage conditions were still being investigated and optimized. This led to the initial definition of rather stringent premature storage conditions, creating significant challenges for vaccine transport and deployment. With more data emerging from the ongoing stability studies, storage conditions and shelf-life periods are adjusted accordingly.

During the preclinical and clinical development phases of vaccine candidates, the manufacturing process obviously does not yet exist in its final form but is still in development. Therefore, depending on the development stage reached, the extent and the informative value of the available quality-relevant data may still be limited in some areas. For example, from a regulatory point of view, a complete validation of the manufacturing process or of control test methods is not expected during a phase I clinical study, neither is a final and complete characterization of the active ingredient including all specifications at this point. Nevertheless, before entering the clinical study phase, it must be ensured that the clinical trial material is sufficiently characterized and has been produced using a defined and consistent manufacturing process. This is examined in detail by the Paul-Ehrlich-Institut as part of the approval procedure for the clinical trial. The specifications of the active ingredient and of the final product must be stated and justified, but preliminary acceptance criteria, which are subsequently adjusted and narrowed in the course of further development, are accepted. Furthermore, it is not expected that the stability data for the active ingredient and the final product have been completed at the beginning of the clinical trial. Instead, interim data are accepted if they can prove the initially intended shelf life of the product to be used in the clinical trial. 

These quality requirements, which are adapted to the respective development status, apply to all vaccine candidates regardless of the disease to be prevented and are laid down in the GCP directive. This regulation also regulates all other regulatory requirements for the approval of clinical studies in Germany. In the case of the SARS-CoV-2 vaccines, no further content adjustments, restrictions, or exceptions to the required quality profile were made. Instead, the shortening and consolidation of the operational processes provide flexibility in terms of time required for the approval process. In this particular situation, it is possible to submit certain parts of the quality documentation later than usual, i.e., during the ongoing assessment of the study. This applies, for example, to certain certificates of analysis for the clinical study material, which can be supplied during the procedure, but must be available and checked before the first test subject in the clinical trial can be vaccinated with this material. For the conduct of the licensing procedure for COVID-19 vaccines, the “Rolling Review” approach has been applied: this is a very flexible and time-optimized mode of processing and assessment of individual data packages immediately upon their availability. In contrast, commonly, only complete dossiers are accepted for regulatory evaluation.

## 3. Requirements for Non-Clinical Examination

The primary goal of preclinical investigations in animal models is to identify and assess vaccine-specific tolerability and efficacy profiles even before clinical trials are started. The preclinical investigations in relevant animal models for specific risk identification and minimization, as well as for testing and deriving appropriate vaccination schemes, are therefore of central importance. The regulatory requirements provide for a clearly defined experimental test program to determine specific pharmacological and toxicological properties of vaccines, which are defined in an extensive regulatory framework of the WHO [7]. As part of these investigations, studies of the primary pharmacological effects have to be carried out, on the basis of which vaccine-specific dose–effect relationships (pharmacodynamics) can be identified and defined. This is one of the bases for the development of the first indications for dose finding and the establishment of a suitable vaccination schedule for later use in humans. In contrast to traditional drugs, which are applied repeatedly and over long periods of time, typically no data on pharmacokinetics, accumulation, and biodistribution have to be collected for vaccines, which are usually administered with few doses and relatively small amounts of substance. However, in the case of live attenuated and replication-competent vector vaccines or for totally novel platform technologies and adjuvants, investigations on the possible distribution and persistence in the body and on the excretion profile (“shedding”) need to be carried out. On a case-by-case regulatory evaluation, such studies have also been required for certain COVID-19 vaccines.

Essential questions regarding the safety of vaccines concern local reactogenicity and systemic toxicity, which have to be determined after single or multiple vaccine administrations. This is done in the context of the so-called “repeat-dose-toxicity” studies, which have to be carried out under the conditions of good laboratory practice (GLP) [8]. These studies are of particular importance for the detection of potential safety risks, since they have to be carried out according to clearly specified criteria and include the determination of important laboratory parameters (e.g., hematology, clinical chemistry) and extensive histological examinations of organ systems in order to reliably identify possible intolerance indicators. If approval for pregnant women is sought, studies of embryo–fetal toxicity are required. In the case of adjuvanted vaccines, significantly more extensive preclinical investigations are required, which include toxicity studies on the adjuvant but also concern the distribution and potential accumulation in the organism. Furthermore, depending on the type of vaccine and the planned indication and application, reproductive, genotoxicity, or carcinogenicity studies may be necessary.

Another central component of preclinical investigations is the analysis of vaccine immunogenicity, i.e., the ability of the vaccine to generate an immune response specifically directed against SARS-CoV-2. This can initially be done using ELISA, immunostaining of antigen-positive cells, or immunoblots in order to obtain information about the antigenicity and to determine the titers of the vaccine-induced, antigen-specific antibodies. These immunogenicity tests also serve to experimentally check and confirm the intended mode of action of the vaccine (“proof of concept”) and are an essential prerequisite for the start of clinical trials. Exposure and protection experiments (“challenge” experiments) are another essential pillar of non-clinical investigations. Since wild-type (wt) mice are not permissive to SARS-CoV-2 infection, hu*ACE2*-transgenic mice are used for COVID-19 vaccine-related studies in the mouse model, which express the human ACE2 receptor on cells and are therefore readily infected with SARS-CoV-2 and develop a clear disease phenotype [9,10]. Recent reports described that serial passaging of SARS-CoV2 in mice results in mouse-adapted SARS-CoV2. Interestingly, this adaption is associated with the N501Y mutation in the spike protein that enables binding to murine ACE2 and infection of mice [11,12].

For all COVID-19 vaccine candidates, relevant data on the induced immune response (e.g., antibody titers, T cell responses, cytokines) must be collected for the intended application regimen (dose strength, number and timing of administration) in a suitable animal model before entering human clinical trials. These studies serve as the basis for the vaccination regimens analyzed in subsequent clinical trials. With regard to preclinical safety trials, the specific requirements are dependent on the product. For COVID-19 vaccine products utilizing established platform technologies of DNA, RNA, or vector vaccines, and for which relevant safety data from animal experiments (in particular, repeat-dose toxicity) for similar platform vaccines are available (e.g., vaccine design utilizes an established technology platform but contains different antigens and is targeted against other infectious diseases), essential parts of pre-clinical testing can take place in parallel to the clinical I/IIa trial. For entirely novel vaccine candidates, the full pre-clinical test program is mandatory before entering clinical phase I.

One potential safety risk in the development of COVID-19 vaccines is the induction of vaccine-induced infection-enhancing antibodies. The so-called ‘antibody-dependent enhancement’ (ADE) was previously observed in animal experiments for SARS-CoV-1- and MERS-specific vaccine candidates [13,14,15,16]. ADE occurs if non-neutralizing antibodies or low-affinity binding antibodies that bind to the pathogen are internalized via cellular Fc-gamma receptors, thus causing an infection of cells that are not normally target cells for the virus. An additional complication that was observed in animal experiments with SARS-CoV-1 and MERS vaccines was enhanced respiratory disease (ERD). Various factors are thought to contribute to the occurrence of ERD. These include a shifted profile of T helper cell (Th) responses towards an enhanced Th2 profile, characterized by the increased expression of various cytokines (e.g. IL-4, IL-5, IL-6, IL-9, IL- 13, and IL-17E (IL-25)), the immigration of eosinophilic cells, and the reprogramming of tissue macrophages in the lungs from a regenerative phenotype to a proinflammatory phenotype, associated with an increase in IL-1, IL-6, IL-8, CXCL-10, and MCP-1 [17,18,19]. Therefore, special attention is paid to these theoretical risks of vaccine-induced disease exacerbation when considering the approval of clinical trials and for the ultimate approval of SARS-CoV-2-specific vaccines. It is therefore a regulatory requirement for both preclinical and clinical investigations that, in order to exclude an increased risk of ADE/ERD, the titers of the induced neutralizing antibodies and other relevant immune parameters (such as T cell responses, cytokine profiles), which may point to a predominantly Th1-based immune response, are examined. The recording of these parameters is of central importance, since suitable animal models (non-human primates (NHP), ferrets, golden hamsters, and transgenic mice) for the reliable investigation and assessment of a potential ADE/ERD risk are currently still under development and are not yet fully qualified for the use as regulation-relevant methods [20,21,22]. On the lines of studies performed on SARS- and MERS-specific vaccines, challenge experiments using NHP could be particularly suitable for detecting an ERD risk. At present, there is no evidence that ADE is involved in immunopathological processes in the context of COVID-19 [23], but the impact of emerging variants on ADE is still unclear.

## 4. Requirements for Clinical Trials

The clinical trials program for the approval of COVID-19 vaccines is based on the disease burden in the population caused by SARS-CoV-2. In Germany, severe courses of disease with hospitalization and deaths after infection with SARS-CoV-2 were mainly observed in adults and the elderly. Therefore, the immunogenicity, efficacy, and safety in this target population has initially been examined as a priority. For the later approval of COVID-19 vaccines for children and adolescents, another specific clinical test program needed to be developed and conducted. For marketing authorization of any human medicinal product, including all COVID-19 vaccine products, in the European Union, sub-mission of a pediatric development plan (PIP) to the EMA Pediatric Committee (PDCO) is obligatory for all manufacturers. This PIP comprises plans for clinical trial protocol specifically designed for the evaluation of the vaccine in the pediatric population.

The regulatory requirements for the clinical development of vaccines are described in international guidelines issued by the WHO [24]. The COVID-19 vaccine testing program follows these guidelines and goes through the phases of clinical development described therein. In the first phase, the safety of experimental COVID-19 vaccines in different doses is closely monitored in a small group of 20–60 healthy, uninfected adults aged 18 to 55. For novel vaccine concepts or for new routes of administration, the vaccine is initially administered to only one or two study participants in a specific dose group in order to minimize the risks. If no serious vaccine side effects have occurred after an observation period specified in the study protocol, the other study participants in this dose group are vaccinated (Guidelines on strategies to identify and mitigate risks for first-in-man and early clinical trials with investigational medicinal products (EMA/CHMP/SWP/28367/07 Rev 1) [25]). In addition to the investigation and documentation of adverse events, the immunogenicity of the various vaccine doses is also checked. For the development of the COVID-19 vaccines, the titer of neutralizing and non-neutralizing antibodies and the cytokine profiles are examined in detail. These investigations are intended to provide an initial indication of the possible immunogenicity as well as a theoretically possible risk of disease exacerbation after vaccination and subsequent exposure to SARS-CoV-2. In all investigations of the antibody response, it is expected that the testing methods used are validated and that an international reference material is used to standardize the results. This makes it possible to compare the immune response evoked by different vaccine candidates.

The dosage that proves to be optimal in terms of tolerability and immunogenicity is checked in the phase 2 clinical trial in a larger study group of several hundred healthy adults. This creates a more robust database for confirming the optimal dosage of the vaccines. In order to collect data at an early stage on the suitability of this dosage for later use in older at-risk patients, the dosage is used in parallel in a group of adults aged 55 and over. The prerequisite for participation is that these test subjects are in stable health with no underlying comorbidities. In this clinical trial as well, the tolerability and immunogenicity of the vaccine candidates are determined. The observation period extends over a period of 6–12 months in order to identify possible vaccine-related side effects that do not occur immediately after vaccination. In addition, it is examined whether and to what extent the neutralizing antibody response decreases over time, since this is considered to be essential for the efficient and lasting protection against infection or disease.

In the phase 3 trials, the efficacy and safety of COVID-19 vaccine candidates are investigated in controlled randomized trials with several thousand subjects aged 18 and over. The aim of this is to prove the prevention of severe courses of disease and/or a laboratory-confirmed SARS-CoV-2 infection in comparison to a control group that does not receive the COVID-19 vaccine. In addition, the neutralizing antibody response is determined in a defined subgroup, which may provide information about the protective antibody titers induced by vaccination. 

In addition, in these phase 3 studies, the occurrence, severity, duration, outcome and frequency of adverse events are determined in comparison to the control group, regardless of causality. Due to the number of subjects included, rarer adverse events can also be detected in these trials.

Whether proof of efficacy is successful in one or more clinical phase 3 studies depends on the infection rate at the time the clinical trials are performed. The optimal study design for proof of effectiveness is currently being discussed internationally. The WHO has published a draft study protocol for such a global effectiveness study, the “WHO solidarity trial”). The WHO encourages different vaccine products to be tested in parallel in different countries and study centers. The chances of direct proof of efficacy are significantly higher if people with an increased risk of infection are included in the studies or if the studies are carried out in regions with high numbers of infections. In the case of low local infection rates or locally unpredictable infection events, direct proof of effectiveness may not be possible. When planning the effectiveness studies, a defined infection or disease rate in the study population must be taken into consideration in order to determine the optimal number of test subjects to achieve statistical proof of effectiveness with a defined test strength during the planned duration of the study. If the frequency of infections or illness in the control group is too low during the study period, no valid statement can be made about the effectiveness within the study population specified in advance. On the other hand, if the number of infections is expected to be very low, the study population cannot automatically be expanded to hundreds of thousands of test subjects, since the implementation of controlled randomized studies on this scale is not feasible from the pure logistic point of view.

If it is possible to define immune correlates, for example a defined neutralizing antibody titer required for protection against infection, the effectiveness of vaccine candidates could also be determined indirectly via immunogenicity testing. All vaccinated individuals who achieve a certain neutralizing antibody titer would therefore be protected. The determination of this correlate of protection could be a result of the studies with convalescent plasma. In addition, this correlate of protection could be verified by stress tests in a suitable animal model.

The documentation and notification obligations of the sponsor of a clinical trial with regard to the safety of the trial subjects are specified in the “Ordinance on the Application of Good Clinical Practice in the Conduct of Clinical Trials with Medicinal Products for Use in Humans” (GCP Ordinance (GCP-V)). Pursuant to this ordinance, the sponsor has to document in detail all adverse events reported to him by the investigators. These records should be sent to the Paul-Ehrlich-Institut upon request in pseudonymized form.

In addition, the sponsor has to inform the responsible ethics committee and the Paul-Ehrlich-Institut about every suspected case of an unexpected serious side effect (“serious unexpected suspected adverse reaction”, SUSAR), immediately and no later than 7 or 15 days after becoming known. This applies to SUSARs from all clinical trials with vaccines under investigation and also to clinical trials outside of Germany, provided that the same vaccine is being tested. The SUSAR reports are recorded in a database at the Paul-Ehrlich-Institut and assessed by a doctor with regard to causality. The obligation to report SUSARs also applies after the clinical trial has ended. If necessary, the Paul-Ehrlich-Institut requests further information and cumulative evaluations in order to check whether the report could have changed the benefit and risk of the study or whether additional risk-minimizing measures, such as dose reduction or additional diagnostics, need to be implemented in order to ensure the safety of the study participants. In addition to evaluating each individual report, the Paul-Ehrlich-Institut regularly conducts weekly statistical evaluations of the cumulative reports.

The sponsor will inform the Paul-Ehrlich-Institut and the responsible ethics committee immediately and no later than 15 days after becoming aware of any issues that require a renewed review of the risk–benefit assessment of the investigated medicinal product. For example, these include:

1. individual reports of expected serious adverse side effects with an unexpected outcome,
2. a clinically relevant increase in the frequency of expected serious adverse side effects,
3. Events connected to the conduct of the study or the development of the investigated medicinal product that could potentially affect the safety of the persons concerned.

The Paul-Ehrlich-Institut can request a list of all suspected serious side effects that occurred during the test as well as a report on the safety of the test participants, which otherwise has to be submitted annually. Overall, the legal regulations ensure close and detailed monitoring of the safety profile of the vaccines during the clinical trial phase.

In summary, it can be stated that the clinical development all kinds of COVID-19 vaccines will go through all phases of regular vaccine development and that, at the time of approval, a sufficient database will be available to adequately assess quality, effectiveness, and safety. Nevertheless, after approval, further studies on the effectiveness (“effectiveness in everyday use”) and safety of the vaccines will also be carried out by the federal authorities in order to ensure that the vaccines retain their positive benefit–risk profile after approval in their intended usage in broad sections of the population. The acceleration of clinical development results from the reduced time required, which is greatly shortened by starting subsequent studies immediately after the results from previous studies become available and by carrying out studies in parallel in different countries. A prerequisite for the success of this approach is a close exchange of information between all partners and good international cooperation.

## 5. Marketing Authorization and Administrative Matters

Novel biotechnologically manufactured medicines are generally authorized for marketing by the European Commission. Consequently, four COVID-19 vaccines have received marketing authorization for the EU/EEA, and it is expected that all COVID-19 vaccines will be evaluated in the so-called centralized procedure coordinated by the European Medicines Agency (EMA). The assessment itself is carried out by two teams which are composed separately of experts from two different national drug regulatory agencies (“Rapporteur” and “Co-Rapporteur”). Both teams independently prepare an assessment report based on the documents submitted by the applicant. The assessment reports can be reviewed and commented on by all national authorities and, after discussion in the Committee for Human Medicinal Products (CHMP) of the EMA, are consolidated in one report. Open points are compiled in question lists and have to be addressed by the applicant in three question-and-answer rounds. The given period for the assessment and discussion by the authorities is up to a maximum of 210 days. The time the applicant needs to answer the questions is not counted towards this time.

Upon request, the regulatory assessment time can be shortened from 210 days to 150 days by means of an accelerated assessment. The prerequisite is an unmet medical need in the case of serious illnesses, which can likely be covered by the new medicinal product. Therefore, accelerated evaluation is reserved for medicinal products that have clear advantages over already approved alternatives or are intended for indications for which no medicinal products are available.

Usually, an approval procedure is started based on a complete dossier. In the context of pandemic situations, the regulatory network is able to evaluate products in a “rolling review”. This is based on the repeated submission and evaluation of parts of the authorization dossier without waiting for a finished, complete dossier. The formal evaluation periods shown above no longer exist in this case. So far, all vaccines that intend to enter the European market have entered the “rolling review”.

The European legislation also allows the approval of medicinal products whose database at the time of approval is less comprehensive than usual, and is therefore flexible enough to consider special circumstances. This is possible if the medicinal products are used to treat, prevent, or diagnose diseases that lead to severe disability or are life-threatening. This regulation can also apply to medicinal products that are intended to be used in crisis situations that pose a threat to public health. The prevention of COVID-19 disease, especially in the context of the pandemic declared by the WHO, fulfills this condition. Even if the data are still incomplete, a risk–benefit analysis must still be possible. This “conditional marketing authorization” requires that the existing gaps in the database are closed after approval [26]. For this purpose, the authorization holder commits to fulfill specific obligations that are stipulated in the course of the authorization. In most cases, this refers to conducting or completing clinical trials.

In principle, the legislation restricts the granting of “conditional approval” to cases in which the clinical part of the data is less comprehensive than usual. An exception applies if the drug is to be used in crisis situations against a threat to public health. In the present case of the SARS-CoV-2 pandemic, a medicinal product could therefore also be approved if the pharmaceutical development has not yet been completed in all details. Here, too, the authorization holder must close these gaps in the database after authorization.

This “conditional admission” is valid for one year and can be extended several times. The authorization holder must therefore submit an application for extension every year, which is assessed by the (Co-)Rapporteurs and decided by CHMP. If all conditions are met and the risk–benefit balance can still be assessed as favorable, the “conditional approval” is converted into a normal approval.

In connection with the approval of a COVID-19 vaccine, the “conditional approval” therefore represents a regulatory tool that enables approval and subsequent market access and can be used to oblige the approval holder to generate further data. The type and scope of the requirements are determined in the course of the approval process, e.g., the requirement to collect further clinical data in populations that were not sufficiently represented in the approval studies. Long-term monitoring of the vaccine’s immunogenicity is also an obvious requirement.

The continuous monitoring and characterization of the safety of a vaccine is agreed and laid down in a separate document, the Risk Management Plan (RMP). The RMP is created for every newly approved medicinal product and is a regular component of pharmacovigilance. It does not represent a special requirement within the scope of the “conditional approval”.

The “conditional approval” has to be distinguished from the so-called “authorization in exceptional cases”. The latter is provided in the European legislation for the event that (for objective and verifiable reasons) it is not possible to submit complete documentation on safety and efficacy when used as intended. This can be the case if the disease is too rare or if it is not possible to conduct the necessary clinical trials due to ethical reasons. With this approval variant, it is also possible to impose conditions that serve to re-evaluate the benefit–risk profile. As with the conditional approval, there is an annual reporting obligation of the approval holder. An example of this type of approval is that for a smallpox vaccine. With smallpox transmission no longer occurring, approval was based on effects in animal models in conjunction with safety and immunogenicity data from healthy volunteers.

## Figures and Tables

**Table 1:** COVID-19 vaccines licensed in the EU (as of 02 June 2021).

	Vaccine Name	Marketing Authorization Holder
**mRNA-vaccines**	Comirnaty	BioNTech Manufacturing GmbH
	COVID-19 Vaccine Moderna	Moderna Biotech Spain, S.L.
**Adenoviral vector vaccines**	Vaxzevria	AstraZeneca AB
	COVID-19 Vaccine Janssen	Janssen-Cilag International NV

## Data Availability

Not applicable.
